# Ethical Considerations for the Use of Social Media in the Human Subjects Research Setting

**DOI:** 10.2196/78183

**Published:** 2025-12-10

**Authors:** Tara Coffin

**Affiliations:** 1 Institutional Review Board, WCG Clinical, 5000 Centregreen Way, Suite 200, Cary, NC, 27513, United States, 1 360-252-2418

**Keywords:** human subjects research, privacy, confidentiality, participant rights, institutional review boards, IRBs, social media, ethical challenges, data collection

## Abstract

The integration of social media into human subjects research offers significant opportunities for data collection, disease surveillance, and participant recruitment. However, it also poses a number of ethical challenges. This article evaluates the dual nature of social media as a research tool, highlighting its potential benefits while also addressing concerns about exacerbating health disparities, compromising participant privacy and confidentiality, challenging expectations around participant disclosure, and perpetuating discriminatory practices. By exploring issues related to equity and privacy, this article discusses the implications of digital recruitment and online behavioral advertising, underscoring the vital role of institutional review boards in ensuring ethical standards are upheld. Furthermore, this work proposes key strategies for researchers and regulatory authorities, emphasizing community engagement, transparency, and inclusive recruitment practices. The analysis aims to guide stakeholders in navigating the ethical complexities of digital research, fostering transparency, trust, and accountability in the realm of human subjects research.

## Introduction

In the rapidly evolving landscape of research, social media has emerged as a double-edged sword—a powerful tool that offers unprecedented opportunities and simultaneously poses significant ethical challenges. The transformative impact of social media on human subjects research is undeniable; it revolutionizes data collection, enhances disease tracking, and simplifies participant recruitment. Yet, beneath these benefits lie crucial ethical dilemmas that demand our attention.

The allure and accessibility of social media for research purposes comes with the potential to deepen existing health disparities, marginalizing those without internet access or digital literacy. Privacy concerns loom large as the vast amounts of personal data shared online raise questions about consent and the risk of discriminatory practices. Navigating these ethical minefields is essential to ensure the responsible use of digital platforms in research.

This article delves into the complex ethical landscape surrounding social media use in human subjects research. By critically evaluating the ethical dimensions of social media as a research tool, this article aims to provide guidance for researchers, sponsors, and regulatory authorities such as the institutional review board (IRB), ensuring that this powerful tool is used responsibly and for the benefit of all.

## Social Media Use in Human Subjects Research

Social media, for the purposes of this discussion, refers to interactive, web-based platforms that allow users to create, share, and discuss content in real time, with a networked audience. Classic examples of social media include Facebook, Instagram, X (formerly known as Twitter), and Reddit. While platforms such as Google and Yelp incorporate user-generated content, they lack the social networking focus central to our definition and are discussed here only where relevant to research ethics.

Online social media networks can serve as a valuable source of data and be leveraged for participant outreach and recruitment. Social-behavioral research benefits from the wealth of publicly available data on social media platforms, enabling researchers to analyze online interactions to identify patterns, themes, and trends that may not be readily apparent through traditional research methods. In this setting, social media can help identify unmet needs, inform the development of targeted interventions, and shed light on factors that influence health outcomes [1-4].

Disease surveillance, a critical component of public health research, has also benefited from the wealth of data available on social media platforms. Researchers use user-generated content to identify illness outbreaks and track the spread of diseases in real-time [5,6]. For example, Twitter has been leveraged to monitor COVID-19 and influenza-like illnesses by analyzing tweets containing specific keywords and phrases, pairing this qualitative data with geolocation data to pinpoint outbreak hotspots [7]. Similarly, Google search trends and Yelp reviews have been used to detect food-borne illnesses, allowing researchers to identify areas of high risk and inform targeted interventions [8].

Beyond infectious disease monitoring, social media usage patterns have been employed as a proxy measure for mental health surveillance, with changes in online behavior serving as an indicator of depression and secondary trauma [9]. These innovative digital-disease surveillance programs rely on a combination of data sources: user-generated content revealing individuals’ experiences and concerns; usage frequency data providing insights into behavioral patterns; and geolocation data contextualizing these insights within specific geographic areas [6]. By integrating these diverse data streams, researchers can address complex public health problems, such as identifying emerging outbreaks, understanding disease transmission dynamics, and developing effective response strategies.

The process of research recruitment has undergone significant transformation in recent years, with social media platforms emerging as a key channel for reaching potential participants [10-12]. Among the various online outreach strategies, online behavioral advertising stands out as a particularly effective approach, leveraging the vast amounts of user data stored by social media platforms [13]. By partnering with these platforms, researchers can tailor their recruitment efforts with unprecedented precision, targeting individuals who meet specific criteria with tailored advertisements. This is achieved through the platform’s algorithm, which analyzes a user’s online behavior, including their age, sex, occupation, race/ethnicity, education, income, and internet search history, to determine whether they match the researcher’s desired demographics. This targeted approach not only increases the efficiency of research recruitment but may also reduce the costs associated with traditional recruitment methods [14]. Moreover, online behavioral advertising enables researchers to include communities that are difficult to reach through traditional outreach mechanisms [11].

When considering these applications, it is apparent that social media can be a beneficial outreach tool and a novel source of publicly available data. However, these applications also raise some ethical concerns. In the next section of this article, these ethical considerations will be examined considering the role regulatory authorities, investigators, and sponsors can play in addressing these concerns.

## Ethical Considerations of Social Media in the Research Setting

### Equity Considerations

Increasing reliance on social media in the research setting raises important ethical considerations, particularly regarding equity. One of the most pressing concerns is the inherent exclusivity of digital spaces, which can exacerbate existing health disparities. Individuals who lack access to the internet or those with low digital literacy skills may be systematically excluded from participation in research [15,16]. This digital divide can perpetuate the marginalization of already underserved populations and further entrench health inequities.

The demographics of social media platforms can vary significantly, with different platforms attracting distinct user groups [17]. For example, some platforms may largely serve younger, more urban, or more affluent users, while others may attract older, more rural, or more diverse users [18]. If research teams unknowingly limit their recruitment outreach to only one or two platforms, they risk omitting entire segments of the general population, potentially reinforcing existing health disparities. For example, a study that only recruits on Instagram may miss individuals over the age of 40, while a study that only recruits on Facebook may neglect younger adults [18].

Researchers increasing their reliance on social media for research recruitment raises important considerations for the IRB. A key concern is the potential for digital recruitment and online data collection to exacerbate existing health disparities and pose significant justice issues [19]. To mitigate these justice issues, researchers can consider the demographics of each platform and deliberately employ a multifaceted recruitment strategy that incorporates a diverse range of platforms, languages, and outreach approaches. By doing so, they can increase the inclusivity of their research, ensure that marginalized voices are heard, and contribute to a more equitable distribution of health resources and outcomes.

IRBs and regulatory authorities can also consider whether the use of social media for recruitment or data collection purposes will result in equitable selection. They should weigh the benefits of digital recruitment, such as increased efficiency and reach, against the potential for unintentional but unjust exclusion of marginalized populations. In the absence of other outreach mechanisms, the IRB should consider the ethical implications of potentially excluding individuals from representation in research. If this exclusion negatively impacts research benefits, researchers should take steps to mitigate these effects, such as by implementing inclusive recruitment strategies, providing alternative participation options for those without internet access, or conducting additional outreach in underserved communities. By supporting approaches that ensure equitable selection and inclusive participation, regulatory authorities can help ensure that the benefits of research are accessible to all individuals, regardless of their digital skills or access to technology [20].

### Privacy Considerations

The rise of digital surveillance and social media-based research recruitment raises critical concerns about privacy, consent, and the exploitation of online data [5,21]. Digital surveillance for research purposes refers to the systematic monitoring, collection, and analysis of data generated through digital technologies, where the individuals creating this data are essentially research participants. The purpose of digital surveillance in this context is to gather relevant data to support research objectives, such as understanding behavior patterns, health outcomes, and other research questions. It involves the use of various tools and software to observe, record, and securely store information about research participants, with particular attention to ethical considerations, informed consent, and privacy protections.

One of the fundamental issues lies in the ambiguous definition of “privacy” in the digital realm [22]. Traditional public health surveillance programs are designed to collect only health-related data, whereas digital surveillance programs indiscriminately mine social media data, which may include both health and non-health information [6]. Social media blurs the boundaries between public and private spheres, and mining data from social media can inadvertently infringe upon individuals’ autonomy and privacy. Furthermore, there is a stark disconnect between users’ perceptions of online privacy—where social media users believe their data is protected from institutional interference—and the reality of data collection and use practices [23,24]. Social media platforms and online services record and store vast amounts of data, including user-generated content, scrolling behavior, and geolocation information, often without users’ explicit consent or understanding [25].

The IRB plays a crucial role in ensuring that social media-based research meets rigorous ethical standards, particularly regarding privacy and confidentiality. As social media data are increasingly mined for research purposes, it is important for IRBs to review the protocols in place for how online user data will be collected, stored, and analyzed. This includes the use of third-party tools, which may be vulnerable to data breaches, to collect and process social media data [26].

Online, user-generated data should be treated as private, even when it is publicly accessible. This means taking appropriate steps to de-identify data as much as possible, securely store identifiable information, and remove non-research-related content, thereby respecting individuals’ privacy and minimizing potential harm [27]. Research involving online user-generated data can also benefit from exploring strategies for building public trust, such as creating opt-in/opt-out systems for research surveillance programs [28].

IRBs review research protocols to determine if researchers are taking adequate steps to either remove non-research-related information, such as names, usernames, and profile pictures, or implement appropriate measures to protect participant privacy [25]. Geolocation data poses additional risks, as it can be used to identify individuals when combined with other data and compromise their privacy [29]. The IRB may also consider the potential risks of re-identification of de-identified data, especially in the context of social media platforms where users may have multiple accounts or engage in online activities that blur the boundaries between personal and public spheres [30].

It is also important for the IRB to push researchers to implement safeguards, such as data aggregation, anonymization, or masking, to prevent the re-identification of participants. Ultimately, it is essential to reexamine of the concept of privacy in the digital age and develop ethical guidelines that protect users’ rights and interests in the face of increasingly ubiquitous online data collection. These guidelines should reflect an updated understanding of privacy in the digital age, with special consideration for the contextual integrity of online data and explicit disclosure to participants, even in the absence of a formal consent process [24,31].

### Obtaining Informed Consent

These privacy concerns are particularly relevant when considering the feasibility of obtaining traditional informed consent. Despite any presumption of privacy, researchers may access publicly available social media data without direct interaction with, or agreement from, the individuals involved. Traditional informed consent procedures are rarely feasible in large-scale social media research and may be waved in accordance with federal regulations [32]. When evaluating a request for a waiver of consent for research using identifiable or potentially identifiable data, the IRB looks for confirmation that (1) the research meets the criteria for minimal risk, (2) consent cannot reasonably be obtained, and (3) the protocol includes adequate safeguards for participant privacy and confidentiality [32]. While these criteria apply broadly, their ethical interpretation differs meaningfully when comparing social media data to a more traditional data source such as electronic medical records (EMRs).

Research involving EMRs typically operates within a clear regulatory framework provided by the Health Insurance Portability and Accountability Act (HIPAA), which explicitly addresses the secondary use of health information [33]. Patients generally expect their health information to remain confidential and, if used for research, to be protected by institutional oversight. EMRs are collected in the office of a trusted care provider or health care facility with clear regulatory protection. This context suggests to the patient population that their private health data are safe from exploitation. It is within this context that a “waiver of consent” is arguably more at home.

In contrast to research involving EMRs, studies using online social media data may meet the technical criteria for a waiver of consent, but often fall outside the scope of traditional regulations. Existing frameworks do not fully address the nuanced implications of mining data that users have publicly shared for non-research purposes [31]. Social media platforms, for example, foster more relaxed privacy attitudes and habitual self-disclosure among users [24]. However, these behaviors should not be mistaken for informed consent regarding secondary research use.

Importantly, even when content is publicly accessible, users may expect a degree of contextual integrity [31]. In other words, they may assume that the information they post to their social media profile will remain within that space and not be repurposed by outside organizations or researchers. This distinction highlights the need for careful ethical consideration. Thus, it is important for IRBs and researchers alike to assess the context of data collection, the nature of the information being used, and the potential risks or impacts on individuals whose data are included [24,31]. These nuances in privacy perception alongside disclosure behaviors challenge the assumption that online social media data is inherently exempt from consent considerations [24,31].

When a waiver of consent is appropriate, researchers should still consider alternative approaches to participant disclosure. Current work emphasizes the importance of transparent research practices and open communication within the context of social media research [34]. This may include (1) public disclosures, (2) opt-out mechanisms, or (3) engagement with community advisory boards, with a focus on fostering trust within online communities [28,35]. Ultimately, the ethical justification for waiving consent in social media research should not rely solely on data accessibility, but must also account for evolving privacy perception, potential harm, and the researcher’s responsibility to uphold public trust.

### Application of Online Behavioral Advertising

The application of online behavioral advertising (OBA) in participant recruitment raises concerns about privacy and equitable selection [36]. OBA services, available through most social media platforms, leverage vast amounts of user data, often collected without individuals’ knowledge or consent [13]. The data are then used to create targeted advertisements, which can be based on sensitive information such as geographic location, income, race/ethnicity, and online activities. Many critics argue that this practice constitutes a privacy infringement, as users are not adequately informed about the data being collected or how it is being used [37]. Other research suggests that OBA invites opportunities for discriminatory targeting, exacerbating existing health disparities and reinforcing harmful stereotypes [38]. While OBA can be an effective recruitment and outreach tool, addressing these issues supports appropriate applications.

Researchers using OBA for participant recruitment should fully disclose their recruitment plans to the IRB for review. Research recruitment, that relies on direct advertisements, is the start of the informed consent process. The IRB is responsible for reviewing these recruitment strategies to ensure that they meet ethical standards. This includes assessing what data will be collected, what targeting variables will be employed, and the potential risks and benefits associated with this approach [39]. The goal of review is to ensure that proposed recruitment does not introduce sampling biases that could impact the validity of the research findings or the rights of participants [40,41].

Partnering with patient advocacy groups to drive outreach can enhance social media recruitment efforts, leveraging established social networks and community trust to promote research opportunities [42]. This approach respects individuals’ privacy and autonomy and fosters more equitable and inclusive research practices. By prioritizing transparency, accountability, and community engagement, researchers can ensure that participant recruitment strategies align with ethical standards and promote the well-being of all individuals involved. Regardless of the researchers’ ability to partner with patient advocacy groups, it is important to support culturally and linguistically responsive recruitment strategies while minimizing the risk of coercion, manipulation, or exploitation of vulnerable individuals. By doing so, researchers can ensure that participant recruitment strategies align with the highest ethical standards and promote the well-being of all individuals involved.

### Recommendations for Responsible Social Media Use in Research

It is important to move from theoretical considerations to actionable steps to ensure social media is leveraged ethically in research. The table below (Table 1) outlines practical measures that researchers, sponsors, and regulatory bodies can take to address some of the ethical concerns unique to the digital environment. These recommendations are intended to serve as a guide for implementing responsible social media research practices that promote inclusivity, safeguard participant privacy and confidentiality, and foster public trust.

**Table 1.** Recommendations for responsible social media use in human subjects research.

Domain	Action point	Description	Example
Recruitment	Assess and diversify social media platforms	Consider user demographics when selecting a social media platform to promote inclusivity	Use both Facebook (older adults) and Instagram (younger adults) for participant outreach
	Promote culturally and linguistically responsive strategies	Adapt messaging and platforms to fit cultural norms and language needs of target audience	Advertise studies in Spanish and English on social media platforms, when enrolling both these populations
	Engage community gatekeepers	Collaborate with advocacy organizations and community groups to improve trust and potential reach	Partner with a local patient advocacy group to share information about the research via their established online social network
	Minimize the use of sensitive targeted advertising	Use OBA^a^ ethically and transparently, avoiding targeting based on sensitive characteristics	Avoid using advertisement mechanisms that target users by race/ethnicity or health status, unless this practice is justified and has received specific IRB^b^ approval
Retention	Support digital literacy	Help participants navigate digital platforms, reducing exclusion due to digital literacy	Provide step-by-step guides to help participants complete study activities
	Develop trust-building measures	Conduct community feedback sessions through the life of the study to build trust in research	Host a question-and-answer session with community members about the study’s use of social media
Data Privacy	Disclosure data collection procedures	Implement public disclosures about research and data use on platforms when possible	Pin a post to a study social media page that outlines data collection practices
	Remove personal information	Take steps to de-identify data collected from social media	Strip profile names and geolocation from data prior to analysis
	Implement privacy safeguards	Securely store and limit access to identifiable online user data	Use encryption for databases storing participant social media data
	Review privacy practices for third-party vendors	If using a third-party vendor for data collection purposes, evaluate the vendor’s data security measures	Choose vendors that are accredited with recognized standards, such as ISO 27001
	Consider contextual privacy expectations	Determine if social media users expect a post to remain within the context of the platform	Do not assume that a publicly available social media post is appropriate to treat as “public” without further consideration

a OBA: online behavioral advertising.

b IRB: institutional review board.

## Conclusions

The use of social media in human subjects research raises important ethical considerations. While social media offers vast opportunities for data collection, disease surveillance, and participant recruitment, it also poses the risks of exacerbating health disparities, infringing on privacy, and perpetuating discrimination. The IRB plays a critical role in ensuring that research studies are conducted in an ethical and responsible manner. To address the risks associated with social media-based research, IRBs should scrutinize recruitment plans, data collection protocols, and privacy safeguards. In tandem, researchers can benefit by prioritizing community engagement to ensure that their recruitment strategies are inclusive, equitable, and respectful of all potential participants. By doing so, we can promote trust, transparency, and accountability in the research enterprise and ensure that social media-based research benefits all the individuals involved.

## Abbreviations

EMR: electronic medical record
HIPAA: Health Insurance Portability and Accountability Act
IRB: institutional review board
OBA: online behavioral advertising
